# The Organisation of a Territorial Army General Hospital

**Published:** 1908-08-22

**Authors:** 


### The Royal Army Medical Corps Section.

### THE ORGANISATION OF A TERRITORIAL ARMY GENERAL HOSPITAL.

Having described in a previous article the arrangements by which it is intended to provide the medical and surgical visiting staff for the general hospitals of the Territorial Army, it is necessary to explain next the lines on which it is proposed to organise the latter so that they may be ready at very short notice to receive patients. To allow of their doing this efficiently and promptly, it has been decided by the military authorities to provide and train in time of peace the adminstrative staff of these hospitals, together with a proportion of the more specially qualified subordinate personnel. By thus maintaining and training such a permanent nucleus, it is considered that on the outbreak of war the mobilisation of the hospital could be carried out quickly and without confusion, for on this nucleus every department of the hospital would be represented, with the result that on the completion of the hospital personnel to its full number there would be someone connected with every branch of the work to direct the newcomers as to their duties.

The administrative staff and the required proportion of the subordinate personnel forming the permanent nucleus will be furnished by the medical service of the Territorial Army, so that they will be members of the R.A.M.C. (T.). Collectively they will number forty-six, and they will consist of the following details: An administrator (Lieut.-Colonel), a registrar (Major), a Quartermaster (Captain), two warrant officers (Sergeant-Majors), 13 sergeants, 8 corporals, 2 buglers, and 18 privates.

The administrator will be a medical man, and will act as the principal medical officer of the hospital. His duties will run very much on the lines of those of a superintendent in a civil hospital. He will be responsible for the discipline of the whole establishment, and will settle all the details for the care of the patients, not, however, in any way interfering with their medical or surgical treatment. The nursing arrangements will also be under his direction, and through him the lady superintendent will allot their special duties to the nursing sisters and nurses. All the necessary requisitions for the wants of the hospital must be approved and signed by him, and he will have to satisfy himself as to the clothing, bedding, nursing, and general comfort of the sick, and as to the quality and cooking of the diets and extras. The care of all hospital equipment and stores, the inspection of all patients on their arrival at and their departure from the hospital, and the sanitary condition of the hospital with its immediate surroundings will all come under his special charge—a combination of duties that call for very special qualifications on the part of anyone occupying the post.

The registrar of the general hospital will act practically as secretary to the administrator, and he will be the channel through whom his orders will be issued. He will compile all statistics and will draw up all returns as well as the general reports to be furnished by the administrator, from which reports, however, all particulars about cases or details of treatment will be excluded. Under the registrar will come, too, everything connected with the admission and discharge of patients, and he will be responsible that all the necessary documents connected with each case are not only correctly taken, but are also kept in safe custody. To assist him in these really clerical duties he will have the services of a certain number of clerks, an arrangement which should materially lighten the work.

The duties of the quartermaster attached to a general hospital are of a varied nature. Not only will he have to prepare and submit to the administrator all returns other than those prepared by the registrar, but he will take over charge of the hospital buildings with all their furnishings, equipment, and stores, and will be responsible to the administrator for their proper maintenance and supervision. He will sign the requisitions for all the necessary hospital supplies, and to him also will fall the custody and proper disposal of all articles of diet and extras, of the medical comforts in the steward's stores, and of the medicines, instruments, and appliances in the hospital, both those in use and those in store. Several other minor duties fall to the quartermaster, but sufficient has been said to show that the post is one of considerable responsibility, and necessitates experience of a varied kind. The allocation of the work of the remainder of the hospital personnel will be so made that all branches of the hospital duties will be provided for. The warrant officers (sergeant-majors) will act under the immediate orders of the administrator, and will assist him in all matters pertaining to discipline and the hospital routine, bringing to his notice any irregularities observed. They will keep the roster of duties and will cause all individuals to be warned for their respective tasks, and they will be responsible for the daily order-book, in which all orders affecting the hospital will appear. From among the sergeants some will be chosen to superintend the nursing orderlies in the wards, some will act as stewards, and others as compounders or as clerks. Of the corporals, some will serve as cooks, some as packstore keepers, and others as clerks, while the privates will be distributed as nursing orderlies, as assistant cooks, and as clerks.

From all this it is quite evident that the system of organisation proposed for a Territorial General Hospital differs in detail from that of a civil one, and that it is essential there should be ready for each general hospital a nucleus of personnel maintained and trained in time of peace, so that on the mobilisation or opening of such a hospital in time of invasion there would be no confusion, the visiting medical staff and the nurses, when they enter on their duties, finding everything in working order. To enable this to be done with certainty, the military authorities have very properly insisted on the permanent nucleus of the personnel undergoing a certain amount of annual training. In the case of the administrator, the registrar and the quartermaster this must take the form of attendance for eight days in a military hospital or at a Territorial medical school. By an attendance of eight days is meant a course of eight days' instruction, but the days need not necessarily be consecutive. In the same way 24 attendances at a suitable military or civil hospital, Territorial medical school, or at any other approved institution will constitute an eight-days' course, but there must not be more than three attendances in one day, and they must not be of less than one hour's duration.

The training of the rest of the personnel will also extend over a course of eight days, and will be carried through at either a military hospital or a Territorial medical school, but once in three years they must attend a camp. The nature of the instruction given at the eight-days' course will include (1) nursing, (2) cooking, and (3) clerking, and it will be entirely of a practical nature. It is to ensure this that a further proviso has been made in connection with the clerking section, that in their case their training must be done in camp or in a military hospital.

Speaking generally of the above conditions of training, we consider that they may very fairly be characterised as elastic, and as being framed in a considerate spirit, so as to meet the varying conditions of civilian life. Further, as all the officers and non-commissioned officers and men will receive pay during their training according to their rank, the rates of remuneration being those of the Regular Army, the terms of service may be regarded as liberal. Under these circumstances, and seeing that the Territorial general hospitals are all to be located in large cities or towns, it is hoped that there will be no difficulty in obtaining the permanent nucleus of their personnel.

